# Tailoring the Synergistic Bronsted-Lewis acidic effects in Heteropolyacid catalysts: Applied in Esterification and Transesterification Reactions

**DOI:** 10.1038/srep13764

**Published:** 2015-09-16

**Authors:** Meilin Tao, Lifang Xue, Zhong Sun, Shengtian Wang, Xiaohong Wang, Junyou Shi

**Affiliations:** 1 Key Lab of Polyoxometalate Science of Ministry of Education, Northeast Normal University, Changchun 130024, P. R. China; 2 Wood Material Science and Engineering Key laboratory of Jilin Province, Beihua University, Jilin, 132013, P. R. China

## Abstract

In order to investigate the influences of Lewis metals on acidic properties and catalytic activities, a series of Keggin heteropolyacid (HPA) catalysts, H_n_PW_11_MO_39_ (M = Ti^IV^, Cu^II^, Al^III^, Sn^IV^, Fe^III^, Cr^III^, Zr^IV^ and Zn^II^; for Ti and Zr, the number of oxygen is 40), were prepared and applied in the esterification and transesterification reactions. Only those cations with moderate Lewis acidity had a higher impact. Ti Substituted HPA, H_5_PW_11_TiO_40_, posse lower acid content compared with Ti_x_H_3−4x_PW_12_O_40_ (Ti partial exchanged protons in saturated H_3_PW_12_O_40_), which demonstrated that the Lewis metal as an addenda atom (H_5_PW_11_TiO_40_) was less efficient than those as counter cations (Ti_x_H_3−4x_PW_12_O_40_). On the other hand, the highest conversion reached 92.2% in transesterification and 97.4% in esterification. Meanwhile, a good result was achieved by H_5_PW_11_TiO_40_ in which the total selectivity of DAG and TGA was 96.7%. In addition, calcination treatment to H_5_PW_11_TiO_40_ make it insoluble in water which resulted in a heterogeneous catalyst feasible for reuse.

Heteropolyacids (HPAs) with Keggin structure are friendly acid catalysts for various organic reactions^1^,^2^,^3^,^4^. In this context, Lewis acidic HPAs have been synthesized through partial exchange of protons with metal cations^5^,^6^,^7^. Sn-exchanged Sn_0.75_H_0.25_PW_12_O_40_ has been studied in different reactions including the selective hydrolysis of cellulose to glucose^8^,^9^, cyanosilylation of ketones and aldehydes^10^, esterification or transesterification^11^,^12^, and Friedel-Crafts alkylation reactions^13^. Zhu’s group developed a high active Ag-exchanged HPA (Ag_1_H_2_PW_12_O_40_) for the glycerol esterification with acetic acid^14^. N. Lingaiah *et al.* reported the ZnPW_12_O_40_ catalyst for the synthesis of glycerol carbonate from glycerol and urea, the high efficiency was attributed to the incorporation of Zn^2+^ into the secondary structure of heterpolytungstate^15^. Up to now, only one report had been reported concerning the acidic properties of H_n_PW_11_MO_40_ (M = Ti, Zr and Th)^16^. Other transition metal mono-substituted PW_11_O_39_^7−^ (Lewis metals include Ti^IV^, Cu^II^, Al^III^, Sn^IV^, Fe^III^, Cr^III^, Zr^IV^ and Zn^II^) have rarely been studied systematically. The focus of our paper is to determine how substituted metals affect the acidic property and catalytic activity of H_n_PW_11_MO_39_. Meanwhile, as one of the most studied processes for evaluation of catalytic activity, esterification between alcohols and organic acids is of great interest in academical and industrial fields. In this case, we selected the esterification of glycerol with acetic acid, which also has an environmental and industrial importance in biomass conversion, as a model reaction to investigate the influence of Lewis center on the catalytic activity. In addition, we also studied the effects of Lewis metals on transesterification. Among all, H_5_PW_11_TiO_40_ was the most active, water-tolerant and acid-tolerant HPAs. Calcination treatment to H_5_PW_11_TiO_40_ made it insoluble in water which confirmed its heterogeneous performance in both esterification and transesterification.

Success of this work might clarify the different effects of Lewis metals on total acidity of HPAs and provide more information on how to select proper HPAs according to different requirements.

## Methods

### Material and reagent

All the chemicals were of AR grade, which were obtained commercially and used without further purification. Na_7_PW_11_O_39_ was synthesized according to the ref 17.

### Instrument

Elemental analysis was carried out using a Leeman Plasma Spec (I) ICP-ES and a PE 2400 CHN elemental analyzer. IR spectra (4000–500 cm^−1^) was recorded in KBr discs on a Nicolet Magna 560 IR spectrometer. The IR spectra of adsorbed pyridine (Py-IR) were depicted by subtracting the spectra before and after exposure to pyridine. X-ray diffraction (XRD) patterns of the sample were collected on a Japan Rigaku Dmax 2000 X-ray diffractometer with Cu Kα radiation (λ = 0.154178 nm). SEM micrographs were recorded on a scan electron microscope (XL30 ESEM FEG 25 kV). The concentrations of esters were determined periodically on Shimazu GC-14C fitted with a HP-INNO Wax capillary column (30 m × 0.25 mm) and flame ionization detector^18^. Each of the catalytic reaction was repeated for three times.

### Catalyst preparation

K_5_PW_11_TiO_40_ was synthesized according to the procedure described previously^19^. Firstly, Ti (SO_4_)_2_ solution (6 mmol in 2 M H_2_SO_4_) was added to the aqueous solution of Na_7_PW_11_O_39_ (6 mmol). Then, adjusting the pH of the mixture to 5.6 by NaHCO_3_, followed by adding solid KCl (2.24 g) until white precipitate, K_5_PW_11_TiO_40_, was formed. After that, the precipitate was filtrated and recrystallized with water for three times. Other catalysts were synthesized as following: firstly, 29.4 g (100 mmol) Na_2_WO_4_ and 1.29 g (9.1 mmol) Na_2_HPO_4_ were dissolved in 80 mL deionized water at room temperature under vigorous stirring. Secondly, 20 mL metal salts aqueous solution (12 mmol) (chlorides for Sn; nitrates for Fe, Cr and Zn; sulfate for Al, Cu and ZrO_2_·xH_2_O) was added dropwise to the former solution with continuous stirring. After that, 100 mL deionized water was added additionally. The pH was adjusted to 5.6 by HNO_3_, then KCl (3.39 g) was added until the precipitate formed. This precipitate was filtrated and recrystallized with water for three times giving potassium salt of KPW_11_M (M = Ti^IV^, Cu^II^, Al^III^, Sn^IV^, Fe^III^, Cr^III^, Zr^IV^ and Zn^II^).

2 g potassium salts of KPW_11_M (M = Ti^IV^, Cu^II^, Al^III^, Sn^IV^, Fe^III^, Cr^III^, Zr^IV^ and Zn^II^) were dissolved respectively in 1000 mL deionized water and the potassium cations were replaced by H^+^ using strong-acid cation exchange resins (Type 732, 20 g) for several times to give HPW_11_M, until no K^+^ can be detected by ICP analysis. Then the HPW_11_M solution was rotary evaporated at 50 °C to remove water and give powders. These powders were calcinated for 3 h at 200 °C to obtain insoluble products. The formation of HPW_11_M undergos the following equations^17^:


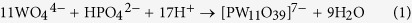



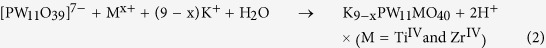



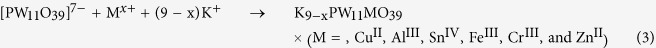



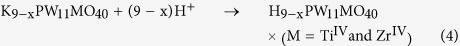



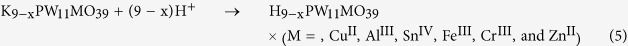


### Determination of the acidic properties

Titration was used to evaluate the total acid content of the solids^20^. 0.05 g HPW_11_M suspended in 45 mL acetonitrile and then the mixture was stirred for 3 h. The density of acid sites in the catalysts was measured by titration with a solution of n-butylamine in acetonitrile (0.05 M) using the indicator anthraquinone (pKa = −8.2). The IR spectra of adsorbed pyridine (Py-IR) helped to measure the acid content and distinguish the properties of acid sites (Lewis or Brønsted). The samples were exposed to the pyridine vapor for 12 h under vacuum (10^−3^ Pa) at 60 °C. The quantification of acidity was calculated by Lambert–Beer equation:


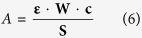


where A is the absorbance (area in cm^−1^), ε is the extinction coefficient (m^2^/mol), W is the sample weight (kg), c is the concentration of acid (mol/kg or mmol/g) and S is the sample disk area (m^2^), respectively. The amount of Brønsted and Lewis acid sites was estimated from the integrated area of the adsorption bands at ca. 1540 and 1450 cm^−1^, respectively, using the extinction coefficient values based on the previous report^21^.

### Esterification reaction

A 25 mL three-necked glass flask equipped with a water-cooled condenser was charged with glycerin (2.3 g, 25 mmol), different volume of acetic acid and certain amount of catalyst. Each mixture was vigorously stirred and reacted at desired temperature for the required reaction time. After the reaction, the mixture was rotary evaporated at 55 °C to remove the excess acetic acid. The products, the unreacted glycerol and the solid catalyst were left in the reactor. Then the catalyst was separated by centrifuging, washed with water, and calcinated at 100 °C for reuse. The liquid mixture was tested by GC. In addition, we have checked the mixture before and after rotary evaporator by GC which confirmed that there was no additional reaction because the time (1 min) was too short to allow any additional reactions occur. The conversion of glycerol and selectivity of glycerides were calculated by the following equations:


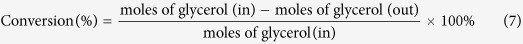



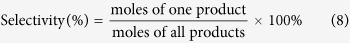


### Transesterification reaction

1.88 mL glycerol triacetin was added to a 25 mL three-necked glass flask with ethanol-cooled condenser (the temperature is −3 °C) under vigorous stirring. After being preheated to 65 °C, the specified amount of methanol (molar ratio of oil/methanol was 1: 6) and 4 wt% of catalyst was added under stirring at 300 rpm to keep the system uniform in temperature and suspension. The reaction was maintained for 4 h at 65 °C and atmospheric pressure. After reaction, the mixture was rotary evaporated at 45 °C to remove the excess methanol, while the products, unreacted triacetin and the solid catalyst were left in the reactor. Then the catalyst was separated by centrifuging, washed with water and calcinated at 100 °C for reuse. The liquid mixture was measured by GC. In addition, we have checked the mixture before and after rotary evaporator by GC which confirmed that there was no additional reaction because the time (1 min) was too short to allow any additional reactions occur.

## Results and Discussion

### Structural characterization of the catalysts

The elemental analyses of HPW_11_M were given in Table 1. These results confirmed the molar ratio of P: W: M = 1:11:1, which showed the formation of mono-metal substituted undeca-tungstophosphates.

**Figure 1:**
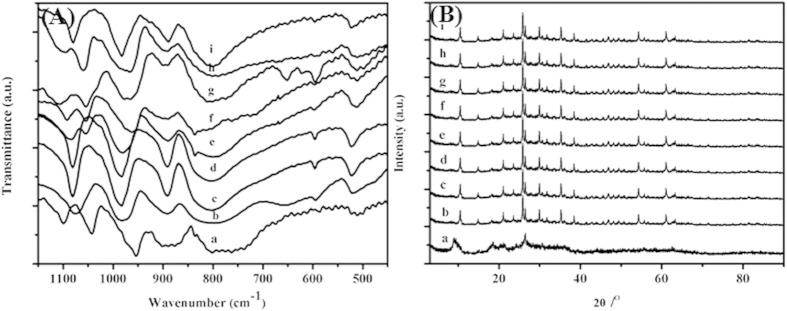
FTIR spectra (A) and XRD patterns (B) of HnPW_11_MO_39_ catalysts. This figure is to confirm the Keggin structure of HnPW_11_MO_39_ catalysts.

Four bands were shown at 1072(8ν_as_ P-O), 977 (ν_as W=O_), 891 (ν_as W−O−W_ inter-octahedral), and 796 cm^−1^ (ν_as W−O−W_ intra-octahedra) in the FTIR spectra of HPW_11_M (Fig. 1A), which were attributed to the stretching vibrational peaks of HPA Keggin anions^22^. Compared with PW_11_O_39_^7−^ (1082, 957, 873, and 763 cm^−1^), some shifts (ν_as W=O_ and ν_as W−O−W_) occurred due to the substitution of W by M ions to form saturated HPAs (Scheme S1). The FTIR spectra of HPW_11_M were similar to that of H_3_PW_12_O_40_ (1080, 982, 890, and 797 cm^−1^), which showed the replacement of tungsten by metal cations formed a dodecatungstophosphoric Keggin structure scussesfully. The IR spectrum gave a band at 580 cm^−1^ which indicated the existence of an M-O bond^23^. The XRD patterns of as-prepared catalysts also supported the results of FTIR (Fig. 1B). It was found that the characteristic peaks were similar to those of PW_11_O_39_^7−^ (10.35, 14.60, 17.85, 20.68, 23.11, 25.44, 31.22, and 34.67°) and only a few changes appeared because of the partial replacement of the W atom by the Ti ion. It demonstrated the successful incorporation of M^n+^ into PW_11_O_39_^7−^ clusters and the formation of good crystals with the Keggin structure.

The morphology of H_5_PW_11_Ti was measured by SEM and EDAX (Fig. S1). It showed that as-prepared material displayed well-shaped crystalline particles with molar ratio of W: P: Ti = 11.07: 1.14: 1.13.

### The acidic properties of the catalysts

**Figure 2:**
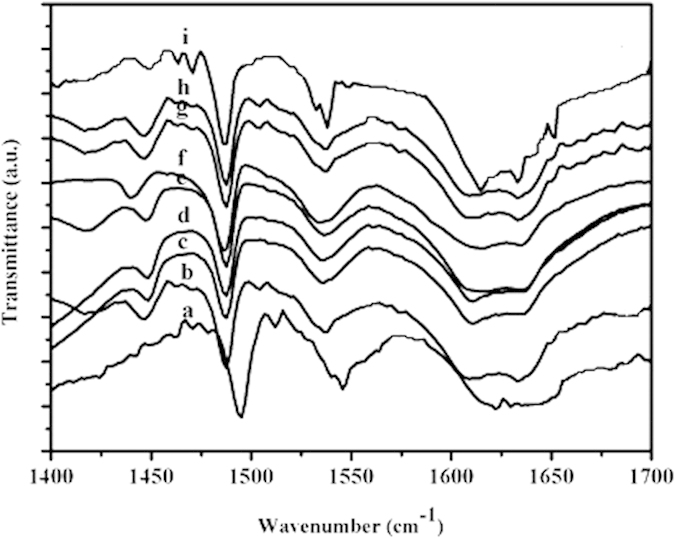
FTIR spectra of pyridine adsorption of H_n_PW_11_MO_39_ catalysts. This figure is a powerful tool for identifying the nature of acid sites, especially to distinguish the lewis acid and Brønsted acid.

The FT-IR spectra of pyridine absorption are a powerful tool for identifying the nature of acid sites^21^. As shown in Fig. 2, HPW_11_M presented typical bands at around 1540 and 1639 cm^−1 ^24 corresponding to strong Brønsted sites. Compared with HPW_11_M, new bands at 1450 and 1610 cm^−1^ were assigned to the coordinated pyridine adsorption on the Lewis acid sites, while the band at 1489 cm^−1^ was originated from the combination of pyridine on both Brønsted and Lewis acid sites^25^. The results indicated that the Lewis acid sites were successfully introduced to HPW_11_M molecules; Therefore, HPW_11_M exhibited double acidic sites including Brønsted sites and Lewis ones.

The total contents of Brønsted acid and Lewis acid were obtained from titration with n-butylamine^20^ and the separated density was calculated from the strength ratio of Brønsted acid and Lewis acid in FT-IR spectra of pyridine absorption^14^ (Table 1). It was found that mono-substituted HPW_11_M gave decreasing trend compared with their parent H_3_PW_12_O_40_ for single Brønsted acid content. In addition, different Lewis metals gave the HPAs with different proton numbers, i.e. the proton numbers were four for Al, Fe and Cr, five for Ti, Cu, Zn and Zr, three for Sn, respectively. For H_5_PW_11_MO_40_, the length of M-O bond decreased as the order of Z/r which is Ti^4+^(7.55) < Zr^4+^(5.55) < Zn^2+^(3.33) < Cu^2+^(3.23). The oxygen attraction of different metals increased in the same order, which might lead to the decrease of proton attraction. Therefore, protons could easily dissociate from HPAs for H_5_PW_11_TiO_40_ and give low value of dissociation constants (the pK_1_). For H_4_PW_11_MO_40_ (Al, Fe, and Cr), the Z/r values were 7.69, 6.12, and 4.84, respectively, which decided the Brønsted acidity in the following sequence: H_4_PW_11_AlO_40_ > H_4_PW_11_FeO_39_ > H_4_PW_11_CrO_39_. Therefore, the metals with higher Lewis acidity such as Ti and Al could give higher Brønsted acidity. Timofeeva^15^ demonstrated some dissociation constants (the pK1) in AcOH as H_5_PW_11_ThO_40_ > H_5_PW_11_ZrO_40_ > H_5_PW_11_TiO_40_ > H_3_PW_12_O_40_ and our results also confirmed these results. At the same time, the Brønsted acidity of H_3_PW_11_SnO_39_ was lower than that of H_3_PW_12_O_40_. By the comparison between H_4_PW_11_MO_40_ (Al, Fe, and Cr) and H_5_PW_11_MO_40_ (Ti, Zr, Cu and Zn), the Brønsted acidity of five protons HPAs was higher than that of four protons. The number of protons played an important role on the Brønsted acidity. For HPAs with the same electron charges in substituent position, the metals with higher Lewis acidity could enhance the Brønsted acidity. Moreover, compared with their parent H_3_PW_12_O_40_, mono-substituted HPAs performed the downward trend for Brønsted acidity no matter how many protons they had. So H_3_PW_12_O_40_ was still the strongest acid among the tested HPAs.

The total acid contents were in the range of H_5_PW_11_TiO_40_ > H_5_PW_11_CuO_39_ > H_3_PW_12_O_40_ > H_3_PW_11_SnO_39_ > H_5_PW_11_ZrO_40_ ~ H_5_PW_11_ZnO_39_ > H_4_PW_11_AlO_40_ > H_4_PW_11_FeO_39_ > H_4_PW_11_CrO_39_. H_5_PW_11_TiO_40_ and H_5_PW_11_CuO_39_ had a higher acid strength than H_3_PW_12_O_40_ which was attributed to the strong combination of Brønsted acid and Lewis acid.

We have reported a series of Ti_x_H_3−x/4_PW_12_O_40_^11^ with higher acidity which was prepared by exchanging the protons of H_3_PW_12_O_40_ with different numbers of Ti^4+^ ions (Scheme S1). The difference of Brønsted acidity between H_5_PW_11_TiO_40_ and Ti_0.25_H_2_PW_12_O_40_ was attributed to their different negative charges of anions, which was −5 for H_5_PW_11_TiO_40_ and −3 for Ti_0.25_H_2_PW_12_O_40_, respectively. Because of the higher number of negative charges, the protons dissociation of H_5_PW_11_TiO_40_ was more difficult compared with Ti_0.25_H_2_PW_12_O_40_. The order of acidic capacity for Ti_x_H_3−x/4_PW_12_O_40_ was Ti_0.6_H_0.6_PW_12_O_40_ (2.886 mol·kg^−1^) > Ti_0.5_H_1_PW_12_O_40_ (2.705 mol·kg^−1^) > Ti_0.25_H_2_PW_12_O_40_ (2.671 mol·kg^−1^) > Ti_0.2_H_2.2_PW_12_O_40_ (2.663 mol·kg^−1^) > Ti_0.1_H_2.6_PW_12_O_40_ (2.614 mol·kg^−1^) > Ti_0.75_PW_12_O_40_ (2.496 mol·kg^−1^) > Ti_0.3_H_1.8_PW_12_O_40_ (2.469 mol·kg^−1^). For Ti_x/4_H_3−x_PW_12_O_40_ series, the strength of acidic property was not incoherent with the number of protons, but was influenced significantly by the number of metals. Ti_0.25_H_2_PW_12_O_40_ gave a higher Brønsted acidity than H_3_PW_12_O_40_, which might be attributed to that Ti shared the attraction force from PW_12_O_40_^3−^ with proton. In this case, proton could easily dissociate from polyanions which would give a higher dissociation constant than H_3_PW_12_O_40_. Therefore, the combination of Ti and H resulted in the higher Brønsted acidity.

### Catalytic activity of HPW_11_M catalysts in esterification

Commonly, Brønsted acid is active mainly in esterification, whereas Lewis acid is more active in transesterification^26^. However, some research showed that Lewis acid is also active in esterification of glycerol. Zhu’s group recently reported a highly-active silver-exchanged phosphotungstic acid catalyst Ag_1_H_2_PW_12_O_40_ which was applied in glycerol esterification with acetic acid, with 96.8% conversion and 48.4, 46.4 and 5.2% selectivity to MAG, DAG and TAG, respectively, and the reaction conditions were 120 °C, 15 min with the molar ratio of 10:1. The high efficiency came from the contribution of Lewis metal Ag^14^. N. Lingaiah’s group also reported the incorporation of Zn into the secondary structure of heteropolytungstate which could promote the conversion of glycerol to carbonate with urea^16^. In order to evaluate the acidic performance of these HPAs, the esterification of glycerol with acetic acid was conducted in the presence of the HPW_11_M catalysts (Table 2). The conversion of glycerol changed as following: H_5_PW_11_TiO_40_ > H_5_PW_11_CuO_39_ > H_3_PW_12_O_40_ > H_3_PW_11_SnO_39_ > H_5_PW_11_ZrO_40_ > H_4_PW_11_FeO_39_ > H_5_PW_11_ZnO_39_ > H_4_PW_11_AlO_40_ > H_4_PW_11_CrO_39_. This order was in accordance with their total acidic density, which suggested that the total acidic strength played an important role on glycerol esterification. The conversions of glycerol for H_5_PW_11_TiO_40_ and H_5_PW_11_CuO_39_ were higher than that of H_3_PW_12_O_40_, which was mainly due to their higher acidic contents.

**Figure 3:**
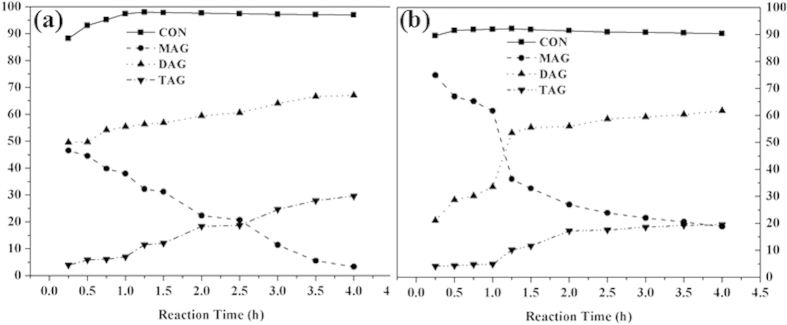
Glycerol conversion and selectivity as a function of reaction time over H_5_PW_11_TiO_40_ and H_3_PW_12_O_40_. Reaction conditions: 75 °C, glycerol/acetic acid = 1:5 (molar ratio), 3 wt% catalyst loading relative to glycerol. This figure is to confirm that extending the reaction time could enhance the selectivities to DAG and TAG which are our target products.

The different catalysts gave different selectivities to glycerol esters. For HPAs with low acidic contents including H_4_PW_11_FeO_39_, H_5_PW_11_ZnO_39_, H_4_PW_11_AlO_39_, H_3_PW_11_SnO_39_, and H_4_PW_11_CrO_39_, the main product was monoacetin whose selectivities were higher than 75%. While for H_3_PW_12_O_40_, H_5_PW_11_TiO_40_, and H_5_PW_11_CuO_39_, the total selectivities to diacetin and triacetin increased. It is known that glycerol esterification with acetic acid is a consecutive reaction including three continuous steps: glycerol + HOAc → MAG + H_2_O; MAG + HOAc → DAG + H_2_O; DAG + HOAc → TAG + H_2_O^14^. It is highly desirable to achieve the maximum production of the valuable DAG and TAG. By now, the selectivities of DAG (58.2%) and TAG (31.9%) reached the highest value within 4 h under the catalysis of Ag_1_H_2_PW_12_O_40_, which was superior or at least comparable to the best catalysts^14^. In our work, the selectivities to DAG and TAG were 55.4 and 6.7% by H_5_PW_11_TiO_40_, and 43.8 and 2.4% by H_5_PW_11_CuO_39_, respectively, which were obtained with the mild conditions : molar ratio of glycerol to acetic acid = 1:5, 75 °C, 3 wt% of catalyst, and 1 h. Extending the reaction time could enhance the selectivities to DAG and TAG (Fig. 3a). It was found that from 0.25 h to 4 h, the selectivity toward MAG obviously decreased to 3.3% while the total selectivity to DAG and TAG increased to 96.7%. For H_5_PW_11_ZrO_40_ and H_5_PW_11_ZnO_39_ with almost the same Lewis acidity, but the former tended to produce more DAG while the later was fond of MAG, respectively. From the point of ionic radii for the two ions, the steric crowding around Lewis acid centers decreased with the increase of ionic radii from Zn^2+^ (0.60 Å) to Zr^4+^ (0.72 Å); therefore, in the same reaction conditions, H_5_PW_11_ZrO_40_ gave more yield of DAG than H_5_PW_11_ZnO_39_.

For H_5_PW_11_TiO_40_ and Ti_0.25_H_2_PW_12_O_40_, the turnover frequencies (TOFs = conversion of glycerol/amount of catalyst (mmol) × time) were 1007.86 and 1055.10 mmol/mmol·h, respectively. The conversion of glycerol was followed the equation obtained by Kozhevnikov *et al.*^27^: log *k* = 1.04*H*_0_ − 3.46, where *k* is expressed as the rate constant, and *H*_0_ is the acidity function of the catalyst solution. Therefore, the catalytic activity of Ti_0.25_H_2_PW_12_O_40_ was higher than that of H_5_PW_11_TiO_40_, which meant that higher Brønsted acidity gave higher glycerol conversion. This was also suitable for the other HPAs substituted by metals. The different selectivities to different esters might be attributed to the pore size distributions of these two catalysts (The pore sizes were 5.62 and 3.83 nm corresponding to H_5_PW_11_TiO_40_ and Ti_0.25_H_2_PW_12_O_40_, respectively). The large size of H_5_PW_11_TiO_40_ permitted higher selectivities to DAG and TAG.

**Figure 4:**
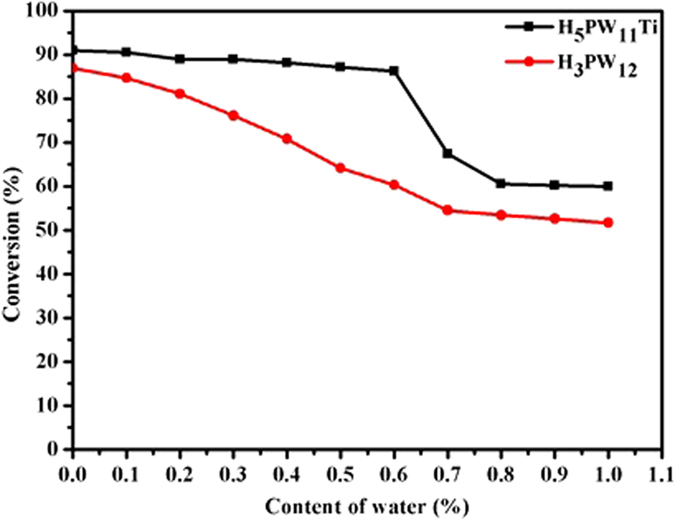
The water-tolerant tests for H_5_PW_11_TiO_40_ and H_3_PW_12_O_40_. Reaction conditions: 75 °C, glycerol/acetic acid = 1: 5 (molar ratio), 3 wt% catalyst loading relative to glycerol, 20 min. This figure is to confirm that when adding extra water to the reaction system, catalytic activity of the two catalysts could be influenced. which meant that H_5_PW_11_TiO_40_ catalyst exhibited a certain water-tolerant property. While H_3_PW_12_O_40_ was not water-tolerant.

For H_3_PW_12_O_40_, the combined selectivities to DAG and TAG reached a maximum value of 81.2% for 4 h. The difference between H_5_PW_11_TiO_40_ and H_3_PW_12_O_40_ was because of their tolerance to water. From the above equations, we could know that water was by-product of the glycerol esterification reaction. Hence, the solid acid catalyst was easily destroyed by water in the system. So a water-tolerant solid acid catalyst was desirable. When adding extra water to the reaction system, catalytic activity of the two catalysts could be influenced (Fig. 4). In view of H_5_PW_11_TiO_40_, the water content ranging from 0 to 0.6 wt% did not play a significant role in the glycerol conversion, which meant that H_5_PW_11_TiO_40_ catalyst exhibited a certain water-tolerant property. On the contrary, the catalytic activity of H_3_PW_12_O_40_ decreased dramatically as the growth of water from 0.1 to 0.6 wt%, which suggested that H_3_PW_12_O_40_ was not water-tolerant. It was reported that if H_2_O molecules were added, they would link with O–H to form O–H····H_2_O^28^,^29^. In the IR spectra of HPAs adsorbing H_2_O (Fig. S2), the peak at 1670 cm^−1^ (corresponding to hydrated OH stretching vibrational peak) belonging to H_5_PW_11_TiO_40_ was bigger than that of H_3_PW_12_O_40_, which indicated that more water molecules could be adsorbed by H_5_PW_11_TiO_40_ than by H_3_PW_12_O_40_. Therefore, the water-tolerance of H_5_PW_11_TiO_40_ was better than that of H_3_PW_12_O_40_. But further increasing the amount of water, the ability of water-tolerance could be destroyed. The different water-tolerance determined that H_3_PW_12_O_40_ could not give the selectivities to DAG and TAG as high as H_5_PW_11_TiO_40_.

Main parameters of the esterification reaction catalyzed by H_5_PW_11_TiO_40_ including temperature, reaction time, catalyst dosage and molar ratio of glycerol to acid were investigated (Fig. S3). It suggested that in order to obtain high yields of DAG and TAG, high molar ratio of glycerol to acid (1:5), long reaction time (4 h), and 75 °C were needed.

### Catalytic activity of HPW_11_M catalysts in transesterification

Based on the suggestion by Santacesaria^30^, Brønsted acid catalysts were active mainly in esterification while Lewis acid catalysts were more active in transesterification. In order to evaluate the efficiency of the HPW_11_M on transesterification, glycerol triacetin was selected to investigate the influence of their Lewis and Brønsted acidity (Table 3). It is known that triglyceride transesterification with methanol is a consecutive reaction including three continuous steps^31^:


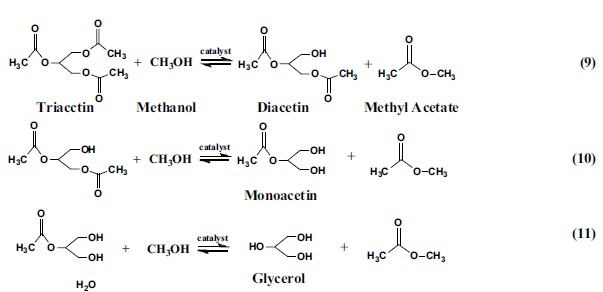


Table 3 showed that conversion of glycerol triacetin changed in the order of Ti_0.25_H_2_PW_12_O_40_ ~ H_3_PW_12_O_40_ > H_5_PW_11_TiO_40_ > H_5_PW_11_CuO_39_ > H_3_PW_11_SnO_39_ > H_5_PW_11_ZrO_40_ ~ H_5_PW_11_ZnO_39_ > H_4_PW_11_AlO_40_ > H_4_PW_11_FeO_39_ > H_4_PW_11_CrO_39_. For TOF, the order was as following: Ti_0.25_H_2_PW_12_O_40_ ~ H_3_PW_12_O_40_ > H_5_PW_11_TiO_40_ > H_5_PW_11_CuO_39_ > H_3_PW_11_SnO_39_ > H_5_PW_11_ZrO_40_ ~ H_5_PW_11_ZnO_39_ > H_4_PW_11_AlO_40_ > H_4_PW_11_FeO_39_ > H_4_PW_11_CrO_39_. The results suggested that HPA catalysts with higher acidity were in favor of high TOF for transesterification of triacetin. The selectivity to glycerol was in the range of Ti_0.25_H_2_PW_12_O_40_ ~ H_3_PW_12_O_40_ > H_5_PW_11_TiO_40_ > H_5_PW_11_CuO_39_ ~ H_4_PW_11_CrO_39_ > H_4_PW_11_FeO_39_ > H_4_PW_11_AlO_40_ > H_5_PW_11_ZnO_39_ > H_5_PW_11_ZrO_40_ > H_3_PW_11_SnO_39_. HPAs with higher Lewis acidity such as Cr, Fe gave higher selectivity to glycerol and tended to promot the conversion of diacetin and monoacetin to glycerol and methyl acetate.

Main parameters of the transesterification reaction catalyzed by H_5_PW_11_TiO_40_ including temperature, reaction time, catalyst dosage and molar ratio of TAG to methanol were investigated (Fig. S4). In order to obtain high yield of glycerol, high molar ratio of triacetin to methanol (1:6), long reaction time (4 h), and 65 °C were needed, with which we have got a 92.2% conversion and a 83.3% selectivity of glycerol, respectively.

It might be more important to convert low quality feedstocks to biodiesel. However, the presence of high FFAs might have adverse effects on the catalyst activity. H_5_PW_11_TiO_40_ might catalyze the esterification of FFA and transeterification of triacetin simultaneously. Therefore, the conversion of triacetin with some FFA contents was catalyzed by H_5_PW_11_TiO_40_ with Lewis acidity and Brønsted one (Fig. S5).

### Reuse of catalysts

**Figure 5:**
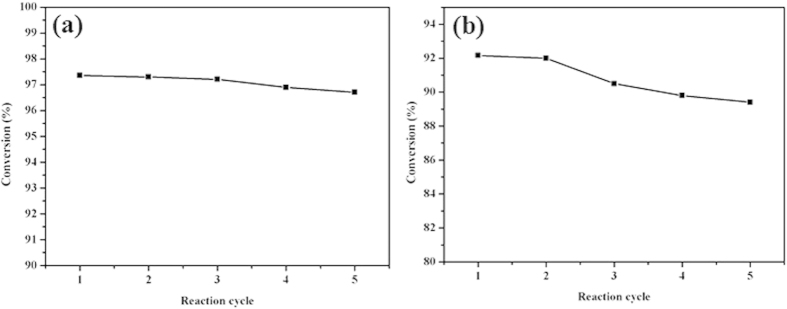
Reusability test preformed for H_5_PW_11_TiO_40_ in esterification and transesterification. This figure is to confirm that H_5_PW_11_TiO_40_ still performed high activity after five reaction cycles either in esterification or transesterification.

It is necessary to determine the nature of H_5_PW_11_TiO_40_ in esterification or transeterification. The solubility of H_5_PW_11_TiO_40_ in acetic acid and methanol was measured by Uv-Vis spectroscopy (Fig. S6). We could see that H_5_PW_11_TiO_40_ was insoluble either in acetic acid or in methanol, which suggested that it performed as a heterogeneous catalyst in both esterification and transesterification reactions. The behavior was attributed to the calcinations treatment to H_5_PW_11_TiO_40_ at 200 °C for about 3 h to form insoluble powders which were also determined by the SEM image and EDAX (Fig. S1). In other word, the catalyst could be easily separated from the production mixture. The catalyst was separated by centrifuging and decanted from the bottom of the reactor, and then washed with ethanol and dried at 60 °C overnight for further reaction cycles. As shown in Fig. 5, there was no considerable change in the catalytic activity after five reaction cycles either in esterification or transesterification. The IR spectrum of the products in transesterification (Fig. S7) indicate that no characteristic peaks corresponding to H_5_PW_11_TiO_40_ were observed in range of 790 to 1000 cm^−1^, which demonstrated that the leaching of H_5_PW_11_TiO_40_ was negligible. The leaching of H_5_PW_11_TiO_40_ was about 0.10 wt% and 0.12 wt% during esterification and transesterification for one cycle, respectively. To further determine the leaching of H_5_PW_11_TiO_40_, the catalyst was seperated after reacting for 20 min (91.0% of glycerol conversion) and was allowed to react further for over 1 h at the same conditions. The result showed that the conversion of glycerol was only 92.4%, which meant that H_5_PW_11_TiO_40_ acted as a heterogeneous catalyst.

The stability of HPAs during the reaction was determined by IR spectroscopy (Fig. S8). After the reaction, the catalysts still kept its Keggin structure. The peaks at 1072, 977, 891, and 796 cm^−1^ attributing to the characteristic bands of Keggin structure could also be observed. The SEM image of the catalyst after reaction (Fig. S9) showed no-change of the morphology during the reaction. Therefore, HPAs was stable and could be reused at least for five cycles.

## Conclusion

This work demonstrated a variety of HPAs H_n_PW_11_M (M = Ti^IV^, Cu^II^, Al^III^, Sn^IV^, Fe^III^, Cr^III^, Zr^IV^ and Zn^II^) with both Lewis and Brønsted acidity. The influence of these Lewis metals on the acidic strength of dodecatungstates showed that (1) moderated Lewis metals gave more influence on total acidity including Ti and Cu; (2) compared to Ti_x_H_3−4x_PW_12_O_40_, Ti in substituted place played a less important role on total acidity. Their catalytic activities in esterification of glycerol and transeterification of triglycerides were generally followed their acidic properties. Among all these HPAs, H_5_PW_11_TiO_40_ displayed properities like Lewis acid sites, insolubility in polar solvents, water-tolerance, acid-tolerance, and stability which were benefit to its excellent performance. To the best of our knowledge, H_5_PW_11_TiO_40_ gave the highest selectivity to desired DAG and TGA (96.7%) in the esterification of glycerol which was higher than the reported Ag_1_H_2_PW_12_O_40_. Moreover, in transeterification, high conversion of triacetin and high yield of glycerol were obtained by H_5_PW_11_TiO_40_. Furthermore, H_5_PW_11_TiO_40_ did not suffer from leaching and deactivation in five reaction cycles either in esterification or transesterification reactions.

This study provides useful information on Lewis metal substituted HPAs. It could be a promising candidate for the catalytic esterification of glycerol and synthesis of valuable biofuel additives, meanwhile, it could also act as a potential catalyst for production of biodiesel using low quality feedstocks.

## Additional Information

**How to cite this article**: Tao, M. *et al.* Tailoring the Synergistic Bronsted-Lewis acidic effects in Heteropolyacid catalysts: Applied in Esterification and Transesterification Reactions. *Sci. Rep.* **5**, 13764; doi: 10.1038/srep13764 (2015).

## Supplementary Material

Supplementary Information

## Figures and Tables

**Table 1:** The characterization of H_n_PW_11_M.

Catalyst	^a^Elementary results (calculated values in parenthesis)/wt%				^b^Brønsted acidity (mmol/g)	^b^Lewis acidity (mmol/g)	^b^Total acidity (mmol/g)	^c^Total acidity (mmol/g)
	H	P	W	M				
H_3_PW_12_O_40_					1.75	0.03	1.78	1.86
Ti_0.25_H_2_PW_12_O_40_	0.12(0.09)	1.22(1.38)	96.73(98.00)	0.44(0.53)	1.94	0.65	2.59	2.67
H_5_PW_11_TiO_40_	0.18(0.18)	1.13(1.13)	74.04(73.64)	1.71(1.74)	1.59	0.56	2.15	2.20
H_5_PW_11_CuO_39_	0.18(0.18)	1.23(1.13)	73.61(73.65)	2.44(2.31)	1.52	0.41	1.93	1.98
H_3_PW_11_SnO_39_	0.17(0.11)	1.14(1.11)	73.84(72.25)	3.99(4.24)	1.22	0.32	1.54	1.61
H_5_PW_11_ZrO_40_	0.18(0.18)	1.13(1.11)	72.53(72.5)	3.34(3.27)	1.14	0.25	1.39	1.45
H_5_PW_11_ZnO_39_	0.17(0.18)	1.32(1.13)	73.53(73.60)	2.01(2.38)	1.12	0.21	1.33	1.42
H_4_PW_11_AlO_39_	0.15(0.15)	1.11(1.14)	74.11(74.23)	1.04(0.99)	0.59	0.60	1.19	1.21
H_4_PW_11_FeO_39_	0.14(0.15)	1.10(1.13)	73.04(73.88)	1.89(2.04)	0.57	0.58	1.15	1.19
H_4_PW_11_CrO_39_	0.14(0.15)	1.11(1.13)	72.82(73.99)	1.94(1.90)	0.54	0.40	0.94	0.98

^a^The elementary results were calculated by the ICP-ES and a PE 2400 CHN elemental analyzer.

^b^The B, L acidity and total acidity were valued by the IR spectra of adsorbed pyridine and calculated by Lambert-Beer equation.

^c^The total acidity was measured by titration.

**Table 2:** The comparison between different catalysts on esterification reaction^a^.

Catalysts	CON(%)	MAG(%)	DAG(%)	TAG(%)	Totalselectivityto DAG andTAG	TOF^b^(mmol/mmol·h)
H_5_PW_11_TiO_40_	97.4	37.9	55.4	6.7	62.1	1007.86
Ti_0.25_H_2_PW_12_O_40_	95.0	52.5	39.6	7.9	47.5	1055.10
H_5_PW_11_CuO_39_	94.9	54.0	43.6	2.4	46.0	981.88
H_3_PW_12_O_40_	90.9	61.7	33.5	4.8	38.3	977.30
H_3_PW_11_SnO_39_	77.9	76.1	22.9	1.0	23.9	821.61
H_5_PW_11_ZrO_40_	75.3	54.4	34.3	11.4	45.8	791.49
H_5_PW_11_ZnO_39_	73.5	89.8	9.0	1.2	10.2	760.97
H_4_PW_11_AlO_39_	71.9	88.5	11.2	0.3	11.5	733.73
H_4_PW_11_FeO_39_	70.6	84.1	15.4	0.5	15.9	728.15
H_4_PW_11_CrO_39_	66.9	74.8	24.4	0.8	32.4	689.00

^a^Reaction conditions: Molar ratio of glycerol to acetic acid = 1:5, 75 °C, 3 wt% of catalyst, and 60 min.

^b^TOF = n_(Gly)_ × conversion/time·n_(catalyst)_.

**Table 3:** The comparison between different catalysts on transesterification^a^.

catalyst	CON(%)	GLY(%)	MAG(%)	DAG(%)	TOF^b^ (mmol/mmol·h)
Ti_0.25_H_2_PW_12_	99.8	89.1	10.7	0.2	70.69
H_3_PW_12_O_40_	99.2	89.5	10.1	0.4	70.00
H_5_PW_11_TiO_40_	92.2	83.3	12.2	4.5	62.03
H_5_PW_11_CuO_39_	82.3	59.8	26.1	14.1	55.36
H_3_PW_11_SnO_39_	69.3	4.3	43.4	52.3	47.83
H_5_PW_11_ZrO_40_	65.8	8.5	17.5	74.0	44.97
H_5_PW_11_ZnO_39_	64.3	20.7	34.6	44.7	43.29
H_4_PW_11_AlO_39_	44.4	32.5	2.6	64.9	29.63
H_4_PW_11_FeO_39_	36.6	41.7	31.5	26.8	24.54
H_4_PW_11_CrO_39_	20.4	58.2	37.6	4.2	13.66

^a^Reaction conditions: molar ratio of TAG to methanol = 1:6, 65 °C, 4 wt% of catalyst, and 4 h.

^b^TOF = n_(TAG)_ × conversion/time·n_(catalyst)_.
