# Supplementary material for: Tailoring the Synergistic Bronsted-Lewis acidic effects in Heteropolyacid catalysts: Applied in Esterification and Transesterification Reactions
Source: Sci Rep. 2015 Sep 16;5:13764. doi: 10.1038/srep13764 (PMC4570989; doi:10.1038/srep13764)
Supplement: Supplementary Information [file srep13764-s1.doc]

**Tailoring the Synergistic Bronsted-Lewis acidic effects in Heteropolyacid catalysts: Applied in Esterification and Transesterification Reactions**

Meilin Tao, Lifang Xue, Zhong Sun, Shengtian Wang, Xiaohong Wang*, Junyou Shi^2^, *

^1^ Key Lab of Polyoxometalate Science of Ministry of Education, Northeast Normal University, Changchun 130024, P. R. China, E-mail: [wangxh665@nenu.edu.cn](mailto:wangxh665@nenu.edu.cn), Fax: 0086-431-85099759

^2^ Wood Material Science and Engineering Key laboratory of Jilin Province, Beihua University, Jilin, 132013, P. R. China


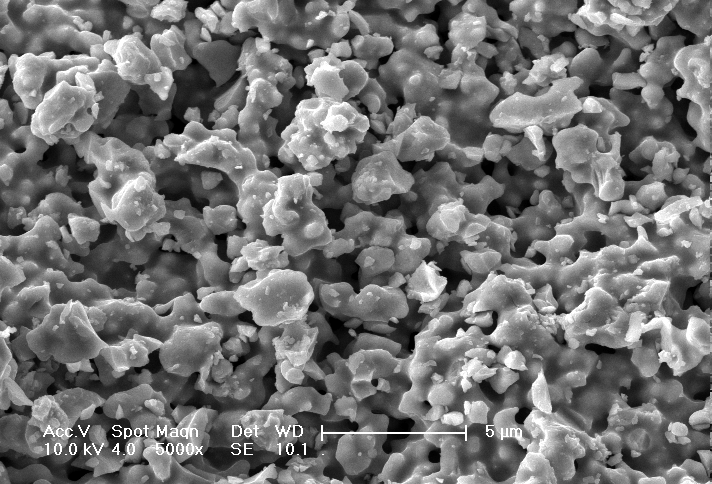




**Fig. S1.** The SEM micrographs (Left) and EDAX diagram (Right) of H_5_PW_11_TiO_40_.


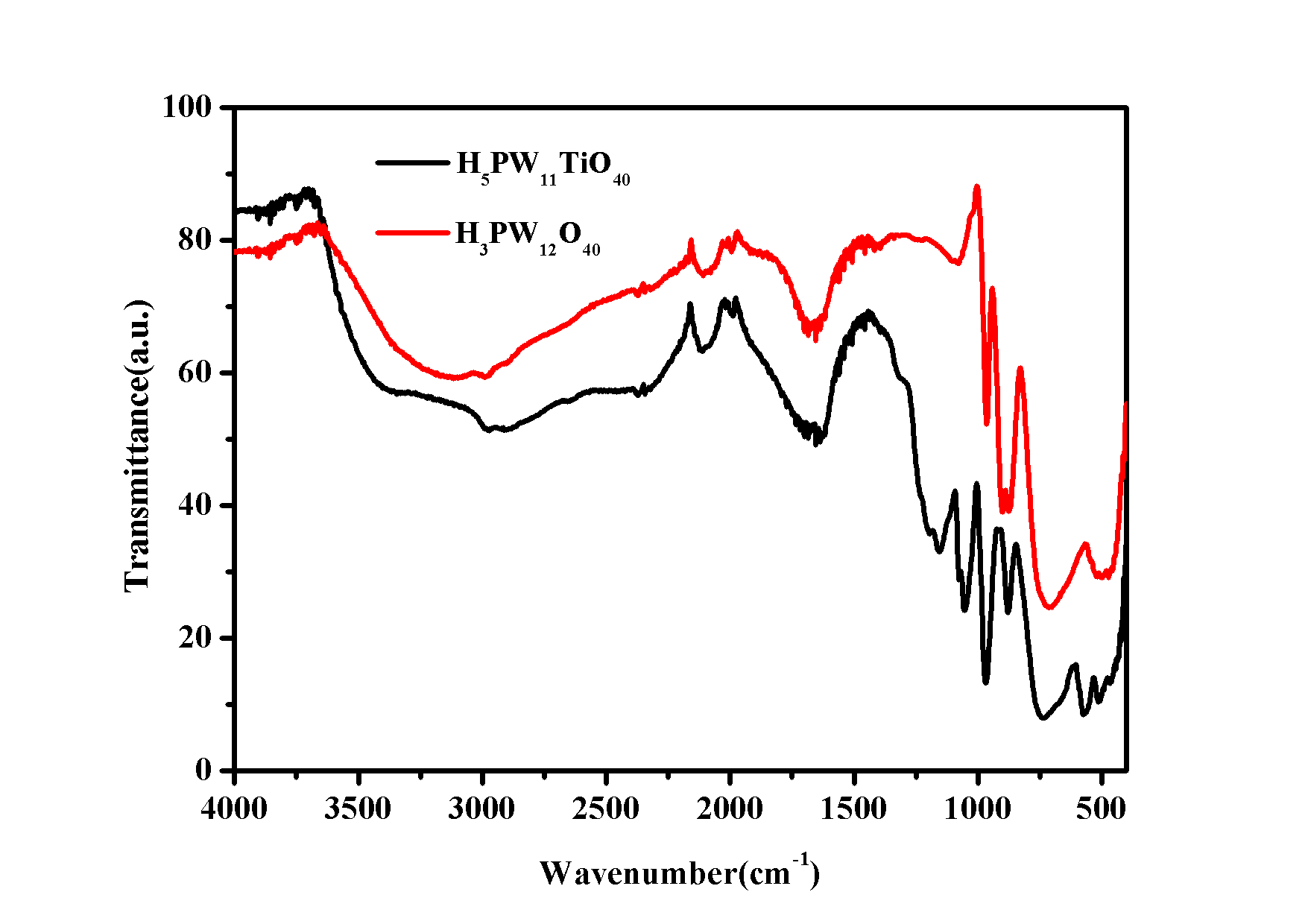


**Fig. S2.** FTIR spectra of H_5_PW_11_TiO_40_ and H_3_PW_12_O_40_ after being dealt with water.


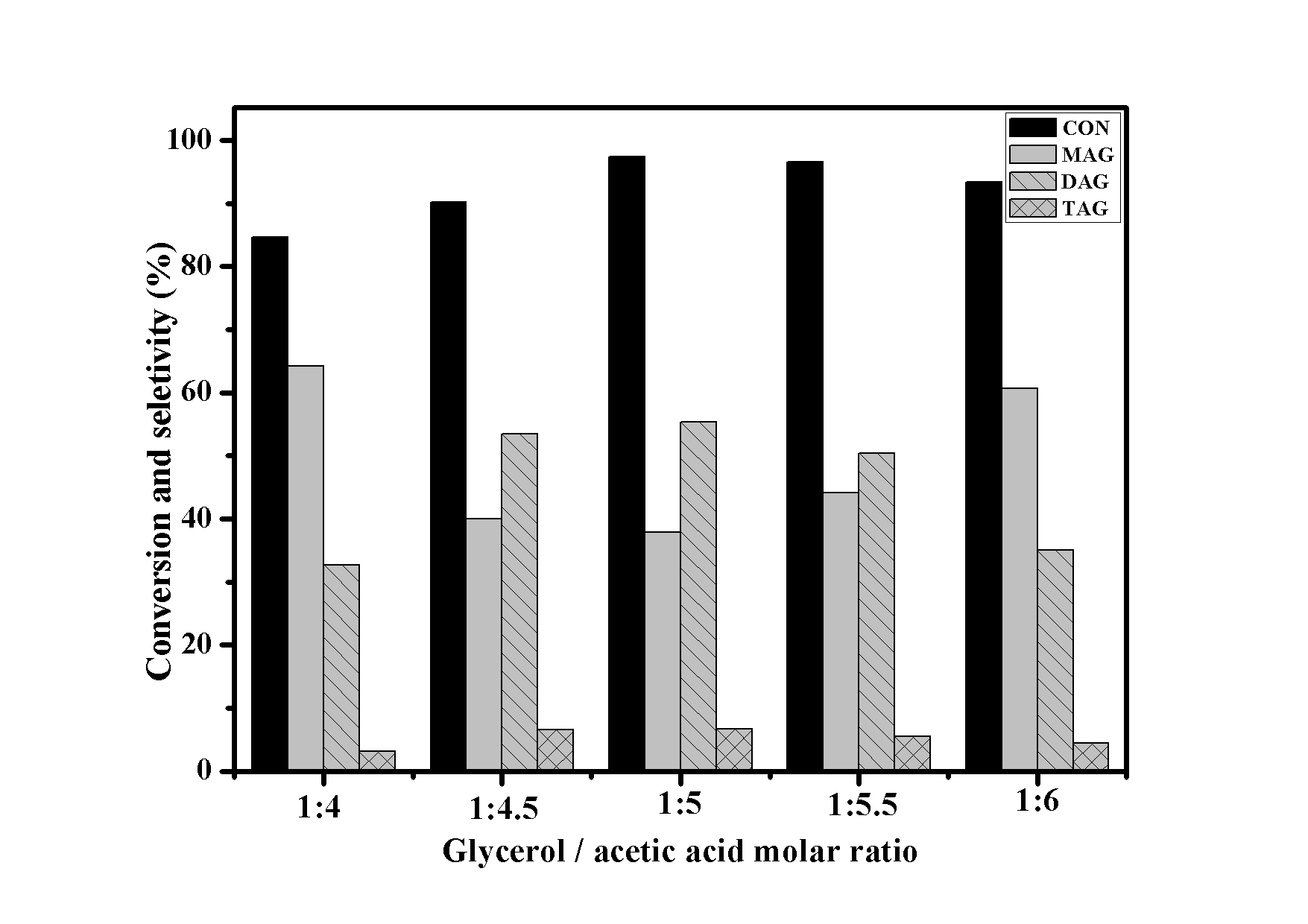

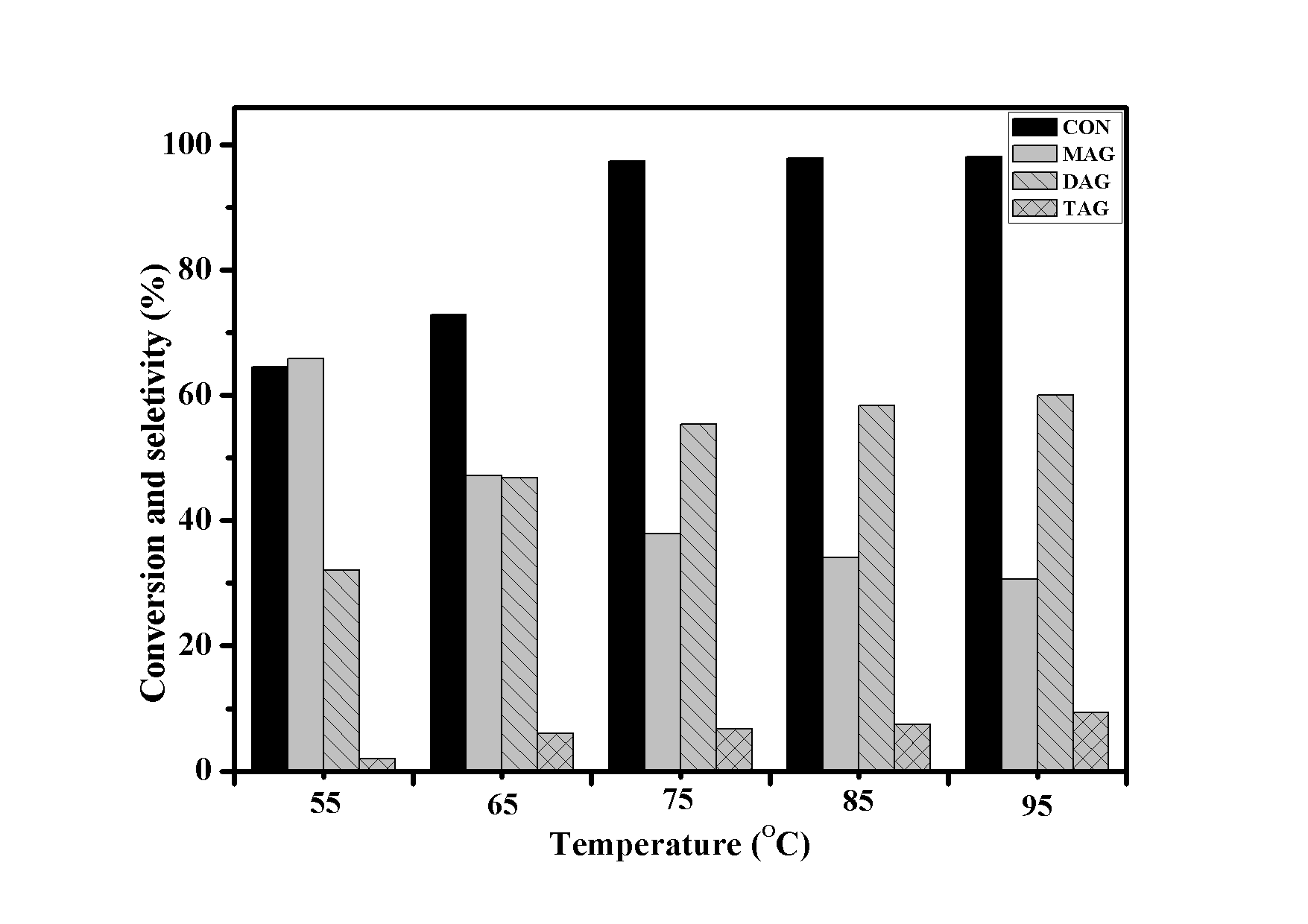

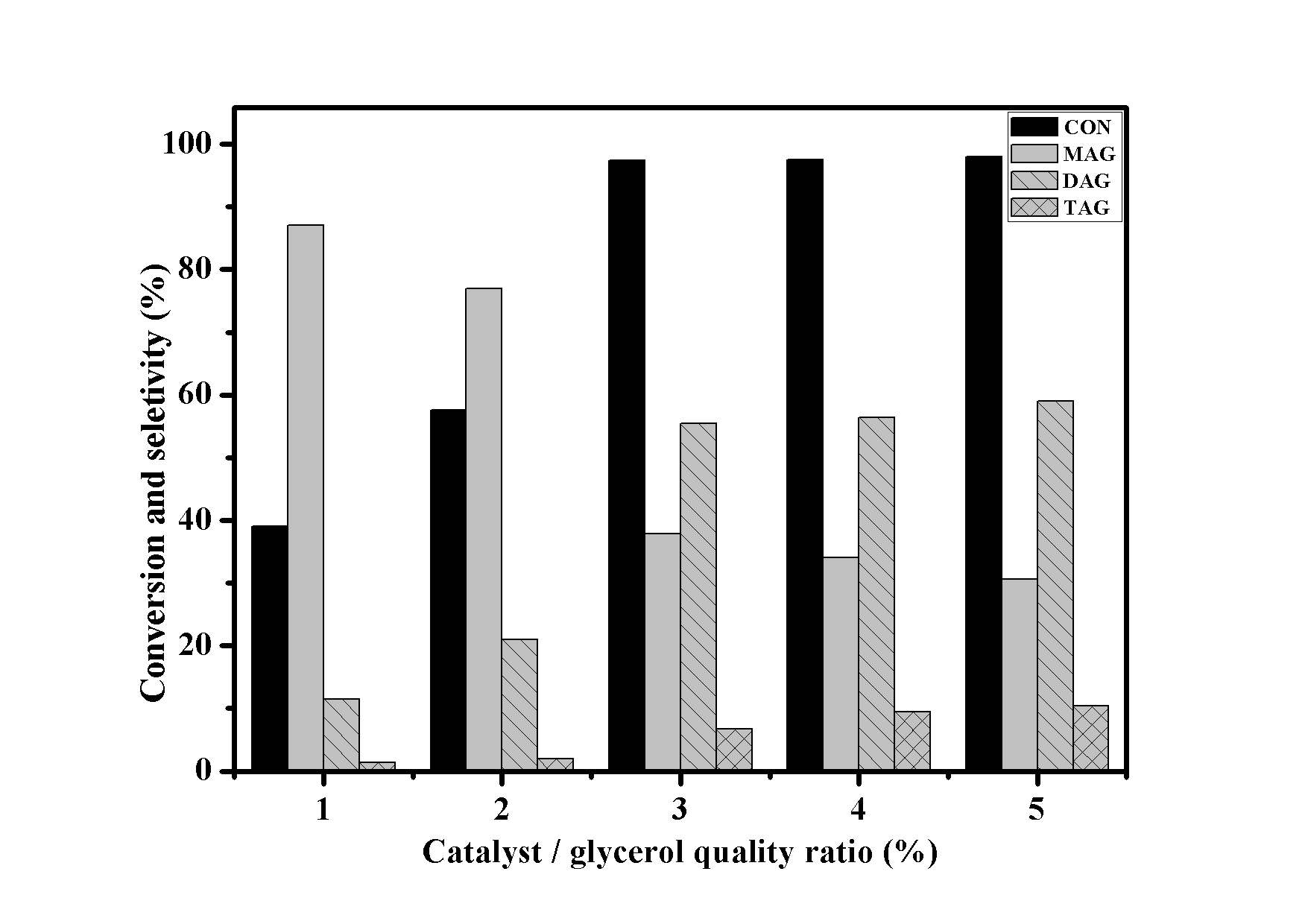


**(a)**

**(b)**

**(c)**

**Fig. S3.** The conversion and selectivity of esterification under conventional process at various process parameters of

1. reaction temperature (molar ratio of acetic acid : glycerol =5:1, 3 wt% catalyst, 1 h);
2. molar ratio of glycerol to acetic acid (75 ºC, 3 wt% of catalyst, 1 h);
3. amount of catalyst (molar ratio of acetic acid : glycerol = 5: 1, 75 ºC, 1 h).


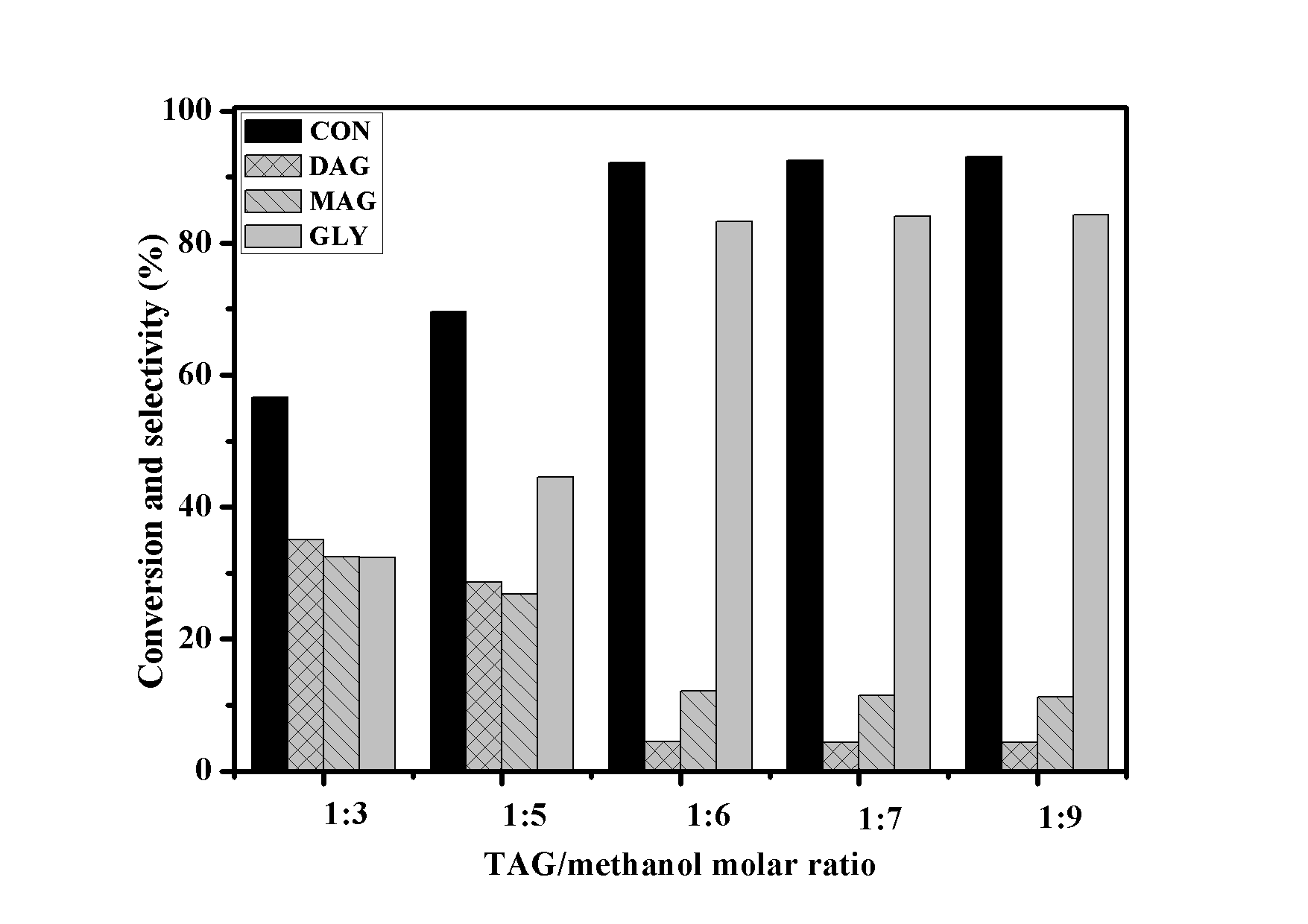

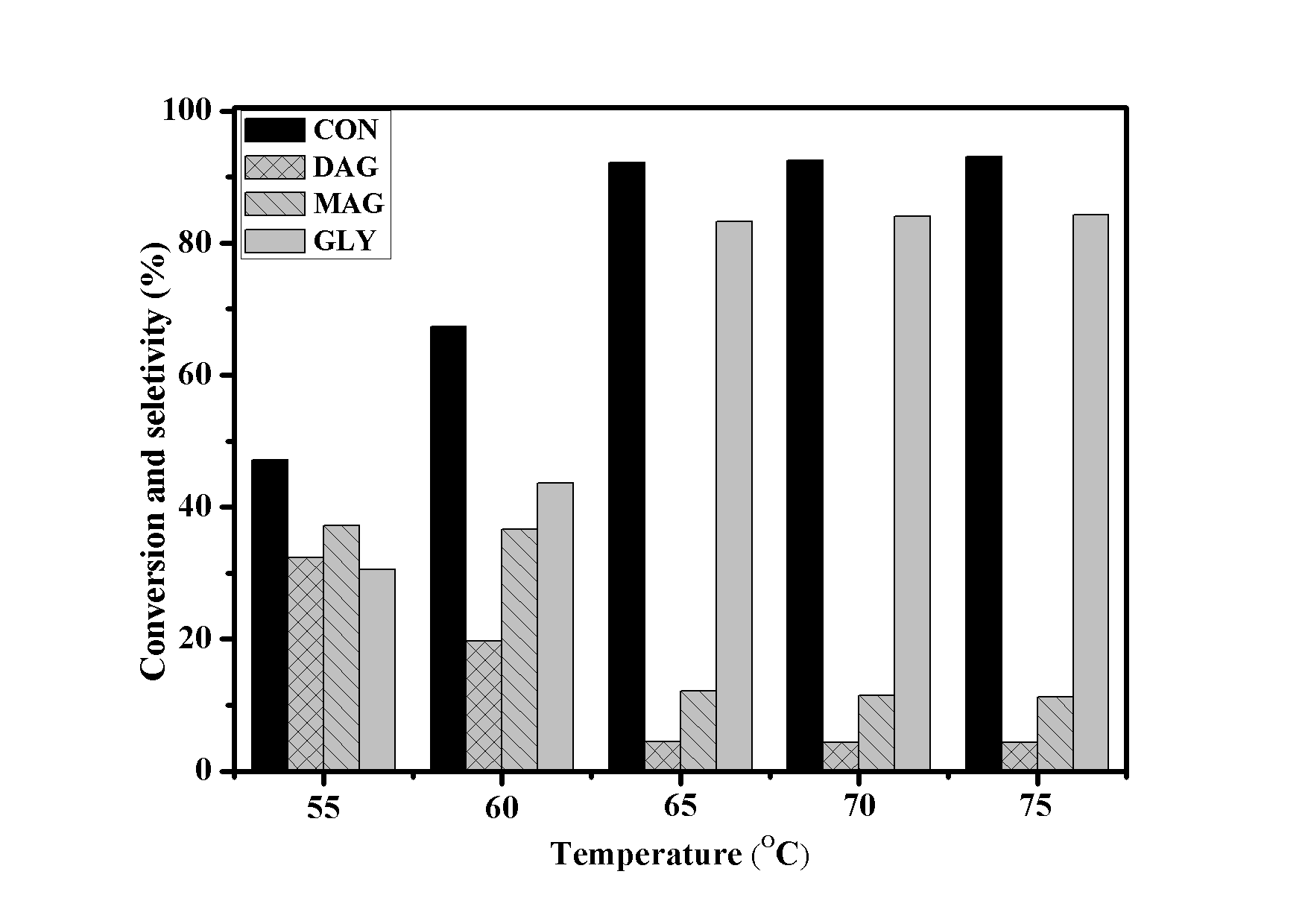

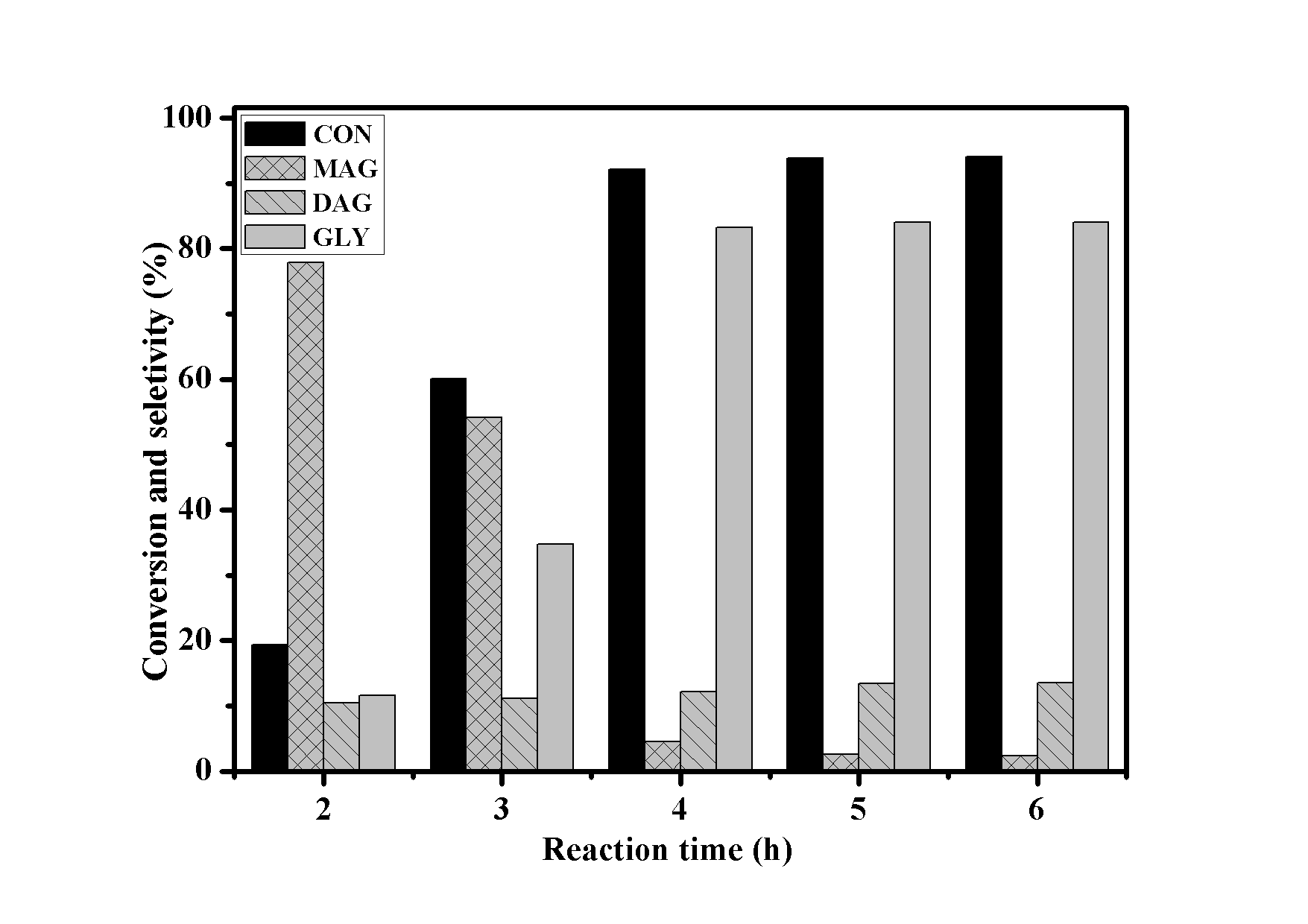

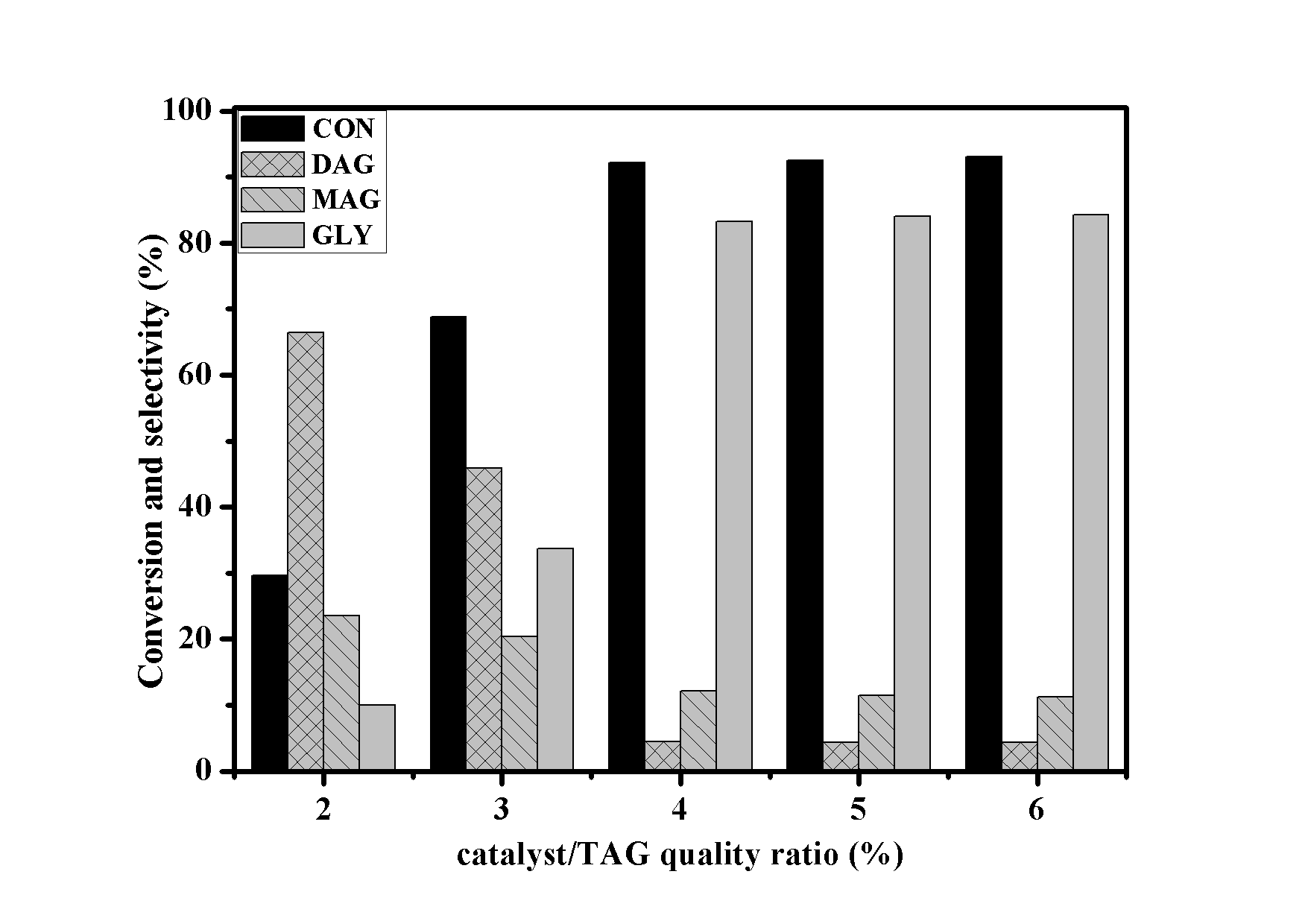


**(d)**

**(a)**

**(b)**

**(c)**

**Fig. S4.** The conversion and selectivity of transesterification under conventional process at various process parameters of

1. reaction time (molar ratio of TAG to methanol = 1:6, 65 ºC, 4 wt% of catalyst);
2. reaction temperature (molar ratio of TAG to methanol = 1:6, 4 wt% of catalyst, 4 h);
3. molar ratio of TAG to methanol (65 ºC, 4 wt% of catalyst, 4 h);
4. amount of catalyst (molar ratio of TAG to methanol = 1:6, 65 ºC, 4 h).

**
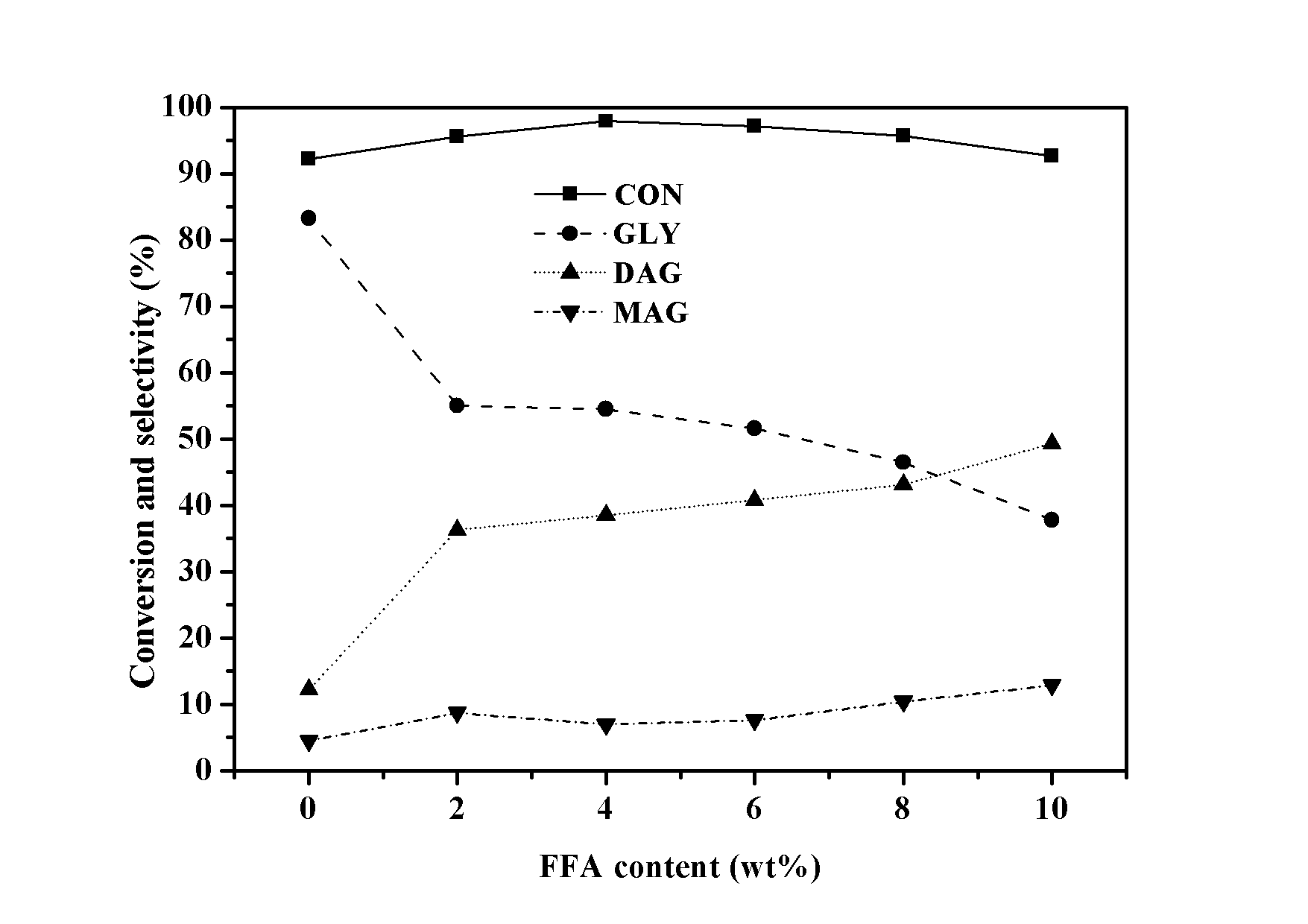
**

**Fig. S5.** The influence of [acid](../../../../C:/Users/Administrator/AppData/Local/Yodao/DeskDict/frame/20140904160431/javascript:void(0)%3B) [content](../../../../C:/Users/Administrator/AppData/Local/Yodao/DeskDict/frame/20140904160431/javascript:void(0)%3B) on transesterification.

Reaction conditions: molar ratio of TAG to methanol = 1:6, 65 ºC, 4 h, 4 wt% of catalyst.







**(a)**

**(b)**

**Fig. S6.** Uv-Vis [spectrum](../../../../C:/Users/Administrator/AppData/Local/Yodao/DeskDict/frame/20140904160431/javascript:void(0)%3B) of H_5_PW_11_TiO_40_ dissolved in acetic acid (a) and methanol (b).





**Fig. S7.** FTIR spectrum of product after transesterification reaction


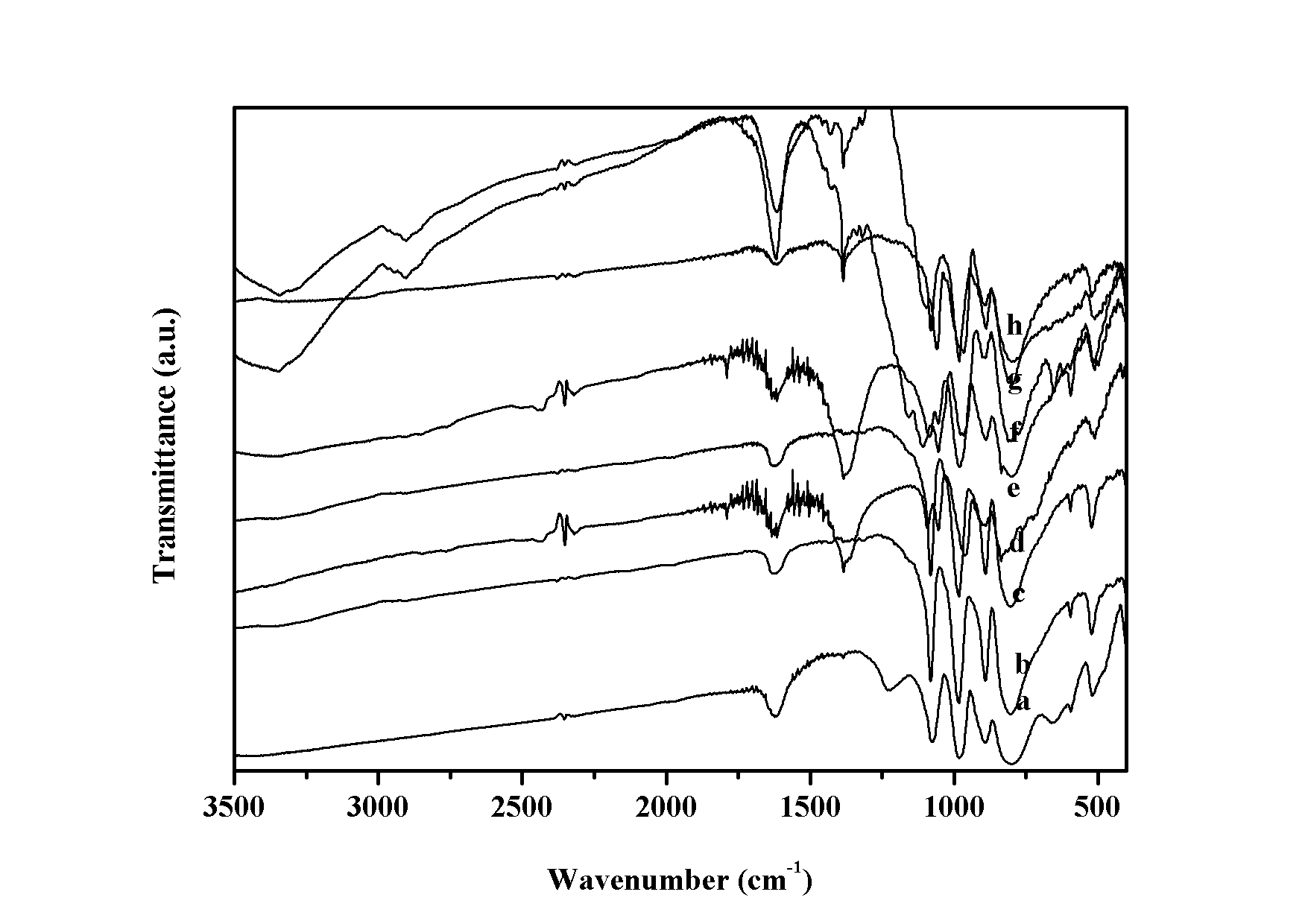

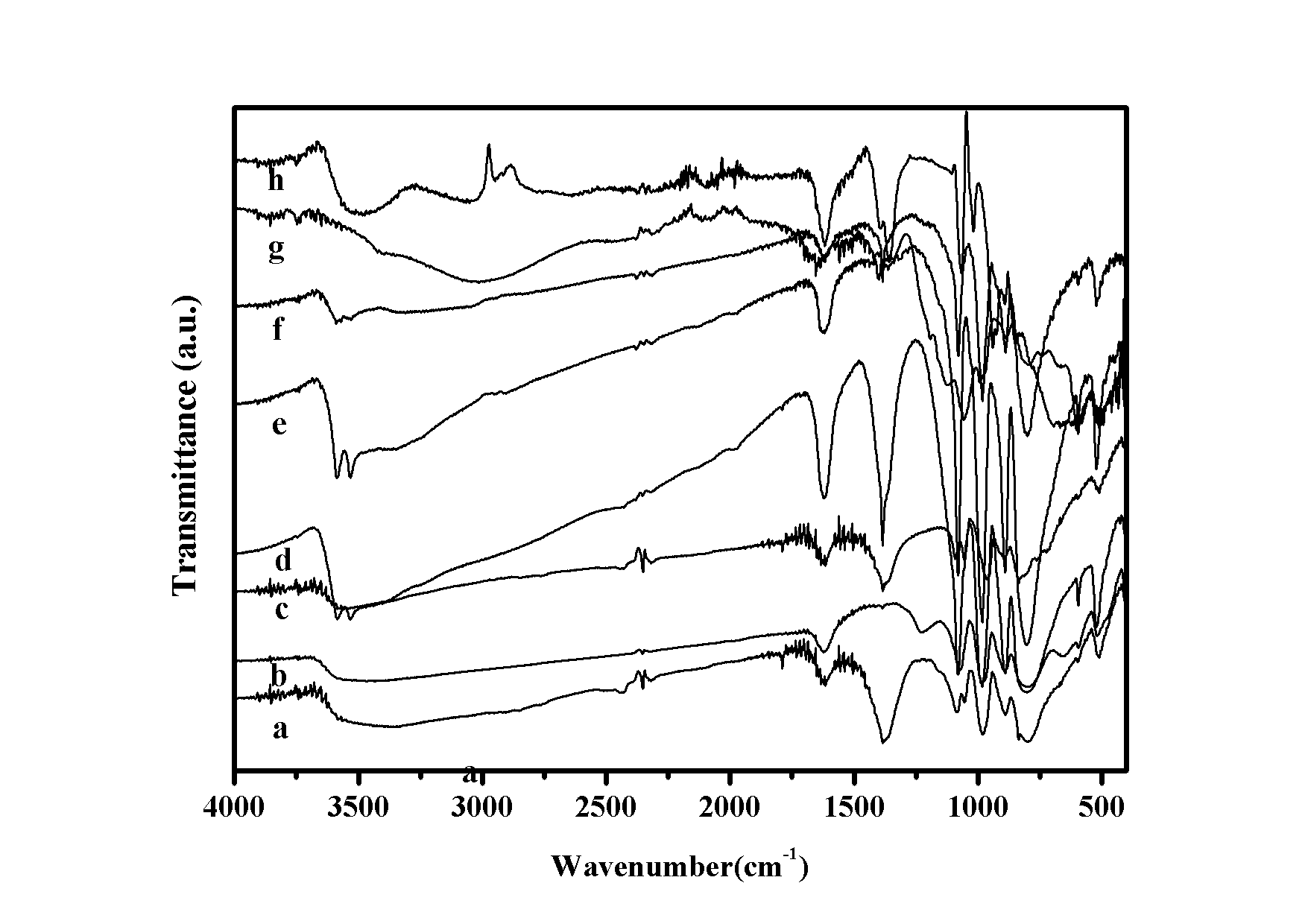


**(A)**

**(B)**

**Fig. S8.** FTIR spectra of catalysts after esterification (A) and transesterification (B).

(a) H_5_PW_11_TiO_40_, (b) H_3_PW_11_SnO_39_, (c) H_4_PW_11_AlO_39_, (d) H_5_PW_11_CuO_39_,

(e) H_4_PW_11_CrO_39_, (f) H_5_PW_11_ZnO_39_, (g) H_5_PW_11_ZrO_40_, and (h) H_4_PW_11_FeO_39_.

**
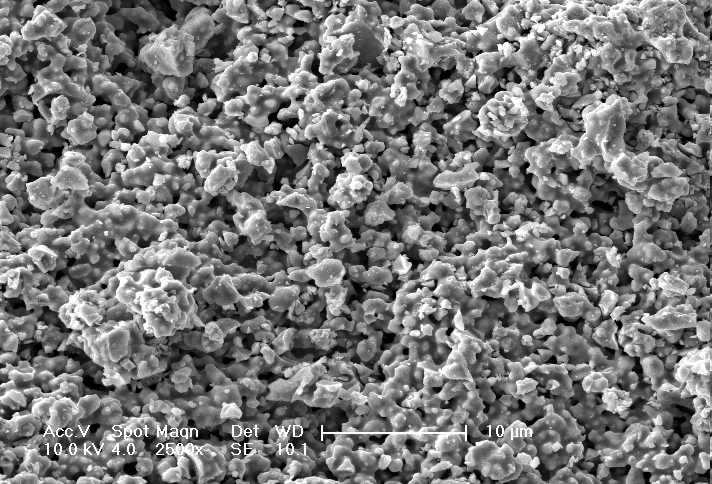
**

**Fig. S9.** The SEM micrographs of H_5_PW_11_TiO_40_ after reaction


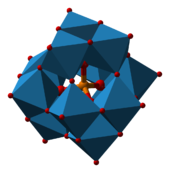


H_3_[ ]


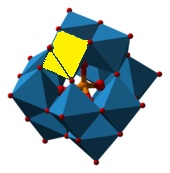


H_5_[ ]

**Ti**

**Scheme S1.** The structure of Keggin polyoxotungstates, H_3_PW_12_O_40_ (left) and H_5_PW_11_TiO_40_ (right).
